# Enhanced Cycling Performance of Spinel LiNi_0.5_Mn_1.5_O_4_ Cathodes through Mg-Mn Hetero-Valent Doping via Microwave Sol-Gel Method

**DOI:** 10.3390/ma17194714

**Published:** 2024-09-25

**Authors:** Mingyin Su, Xiongwen Dong, Xinyi Dai, Bingbing Huang, Min Shen, Teng Xu, Qibin Liu

**Affiliations:** 1 College of Material and Metallurgy, Guizhou University, Guiyang 550025, China; 2 Guizhou Dalong Huicheng New Material Co., Ltd., Tongren 557503, China; 3 Key Laboratory of Modern Manufacturing Technology of Educational Ministry, Guiyang 550025, China

**Keywords:** LiNi_0.5_Mn_1.5_O_4_, hetero-valent doping, high-rate cycling performance, microwave sol-gel method

## Abstract

As a high energy density cathode material, further development of high working voltage spinel LiNi_0.5_Mn_1.5_O_4_ has hindered by its rapid capacity degradation. To address this, a hetero-valent substitution of magnesium for manganese was used to synthesize spinel LiNi0.5Mg_x_Mn_1.5−x_O_4_ (x = 0, 0.03, 0.05) via a microwave sol-gel method. XRD and refined results indicate that such strategy leads to the modification of the 16c interstitial sites. The electrical performance demonstrates that a modest substitution (x = 0.03) significantly improves both rate performance (113.1 mAh/g, charge and discharge at 5 C) and cycling stability (85% capacity retention after 500 cycles at 1 C). A higher substitution level (x = 0.05) markedly improves high-rate cycling performance, achieving 96% capacity retention after 500 cycles at 5 C. It offers tailored solutions for various application needs, including capacity-focused and high-current-rate applications. Furthermore, the stable LiNi_0.5_Mg_0.05_Mn_1.45_O_4_ sample could also serve as an effective coating layer for other electrode materials to enhance their cycling stability.

## 1. Introduction

Energy issues have long been a prominent concern, gradually emerging as a significant obstacle to global economic development. Traditional hydrocarbon fuels, being non-renewable, environmentally harmful, and finite resources, are increasingly inadequate to meet present development demands [1]. Consequently, alternative energy sources like solar, wind, hydroenergy and nuclear power are garnering increased attention due to their renewable nature, sustainability and cost effectiveness. However, these alternatives often exhibit temporal and spatial instability, necessitating efficient conversion and storage prior to utilization [1,2,3]. Thus, rechargeable lithium-ion batteries have emerged as superb energy storage materials and find widespread applications across various sectors, owing to their high energy density and cost efficiency since their introduction by Sony Corporation in the 1990s [4]. Notably, the rapid growth of vehicle electrification, encompassing plug-in hybrid electric vehicles (PHEVs) and pure electric vehicles (EVs), in recent years has heightened the demand for batteries with enhanced energy density, power density and cycle performance [5,6].

The spinel LiNi_0.5_Mn_1.5_O_4_ (LNMO) cathode material boasts a high voltage platform of 4.7 V and an impressive energy density of approximately 640 Wh kg^−1^, surpassing commercially available LiCoO_2_ and LiFePO_4_ cathode materials by 20–30% [1]. Additionally, LNMO’s three-dimensional lithium ion transmission channel facilitates high ionic conductivity, enabling rapid lithium diffusion during electrochemical processes [1]. Moreover, its cobalt-free composition positions LNMO as a promising candidate for next-generation lithium-ion battery materials [7,8]. However, either success or failure of LNMO boils down to the same high working voltage. On the one hand, the high working voltage (4.7 V) brings the high energy density. On the other hand, the Ni^2+^ will oxidize to Ni^4+^ ions during the de-lithiation (at high working voltage), which will exhibit extremely strong activity and catalytic properties with the liquid electrolyte resulting in the capacity fading [9,10,11]. In addition, the migration of transition metals (TM) ions from octahedral sites (16d) to tetrahedral Li sites (8a) and empty octahedral sites (16c) in the de-lithiation state is closely associated with the structural failure and the dissolution of Ni/Mn ions, directly diminishing capacity and lifespan [12,13]. Consequently, numerous studies in recent years have focused on doping modification to enhance the electrochemical stability of LNMO [14,15,16]. With the advancement of research and improvements in testing methods, it has been discovered that doping atoms can enter the 16c interstitial sites in LNMO, and this approach has been utilized to enhance the cycling performance of LNMO [17,18,19]. Magnesium (Mg), as a low cost and extremely abundant element, has been used as an extra doping beyond the theoretical chemical formula (Mg_0.1_−LiNi_0.5_Mn_1.5_O_4_) to such modification [19]. However, substitutions involving elements within LNMO itself, such as Mg divalent substitution to Ni (LiMg_x_Ni_0.5−x_Mn_1.5_O_4_) [20,21,22] or Mg hetero-valent substitution to Mn (LiNi_0.5_Mn_1.5−x_Mg_x_O_4_) [23,24], have not shown similar 16c interstitial sites. In addition, with the widespread use of energy, such as in electric vehicles, there are increased demands on battery performance. These include higher discharge specific capacity, longer service life, faster charging rates (to reduce charging time) and higher discharge rates (to achieve greater output power). Such requirements place greater demands on battery materials. Therefore, it is worth investigating whether such 16c interstitial sites can meet these demands.

In this work, a series of hetero-valent substitutions of magnesium (Mg) for manganese (Mn) in spinel LiNi_0.5_Mg_x_Mn_1.5−x_O_4_ (x = 0, 0.03, 0.05) were synthesized by a microwave sol-gel method. This strategy has resulted in a 16c modification that was not observed with previous Mg-Mn hetero-valent doping. Furthermore, the extent of this modification increases with higher Mg content. The results show that a small amount of Mg-Mn modification enhances rate and low current cycling performance, while a higher Mg content stabilizes the higher rate cycling performance of LNMO, allowing MG5 to maintain a capacity retention rate of about 96% after 500 cycles at a high charge–discharge current density of 5 C. Therefore, different amounts of Mg-Mn hetero-valent modification can provide options for various application environments. Certainly, directly using the stable MG5 as a coating layer for other materials also holds significant promise.

## 2. Experimental Section

### 2.1. Material Synthesis

The spinel LiNi_0.5_Mn_1.5_O_4_ (LNMO) samples without Mg doping was synthesized by a microwave-assisted sol-gel method. First, 10 mmol of Ni(CH_3_COO)_2_·4H_2_O, 30 mmol of Mn(CH_3_COO)_2_·4H_2_O and 21 mmol of LiCH_3_COO were dissolved in 80 mL deionized (DI) water as a mother solution (Li:Ni:Mn molar ratio is 1.05:0.5:1.5), and 40 mmol of citric acid (CA) was dissolved in else 50 mL deionized (DI) water as a chelating agent. After being completely dissolved, the CA solution was (1.25 mL/min) added drop by drop to the mother solution at a stirring speed of 300 r/min. The mixture was subjected to microwave irradiation in a SINEO MAS-II Plus microwave reactor (Shanghai, China) at 600 W. The temperature was varied from 25 to 105 °C in about 5 min, and then reacted for 45 min to form a completely dried green sol-gel, then cooled down to room temperature. The xerogel was calcined at 500 °C in air for 5 h. After being ground, the as-calcined powders were sintered at 900 °C for 10 h (3 °C/min) then cooled down to room temperature, named MG0.

The Mg-doped samples LiNi_0.5_Mg_x_Mn_1.5−x_O_4_ (x = 0.03, 0.05) were synthesized same as above synthetic scheme. Mg(CH_3_COO)_2_·4H_2_O was the Mg source in this article. The final product LiNi_0.5_Mg_x_Mn_1.5−x_O_4_ (x = 0.03, 0.05) were named MG3 and MG5. And the synthesis routes and detailed picture of the microwave sol-gel reaction equipment are shown in Supporting Information (Scheme S1 and Figure S1).

### 2.2. Material Characterization

X-ray diffraction (XRD) data were obtained using a Bruker Advanced D8 XRD diffractometer operating in a reflection mode at 40 kV, Cu Kα radiation over the angular range 10–80° 2θ at 0.03° incremental step. Fourier transform infrared spectroscopy (FT-IR) spectra were collected on a Nicolet 6700 (Thermo Fisher Scientific, Waltham, MA, USA) Spectrometer Frontier instrument in range from 400 to 4000 cm^−1^. X-ray photoelectron spectroscopy (XPS) was performed on a Thermo Scientific K-Alpha instrument (Thermo Fisher Scientific, Waltham, MA, USA). All XPS values were referenced to the adventitious surface C1s peak at 284.6 eV. The morphology of the samples was probed by scanning electron microscopy (SEM, Zeiss Sigma 300, Oberkochen, Germany) and transmission electron microscopy (TEM, JEOL JEM-2010F 200 kV, Akishima-shi, Japan). Energy dispersive spectroscopy (EDS) was also performed using a JEOL ARM-200F (OXFORD X-MAX).

### 2.3. Electrochemical Measurements

LNMO active material (LNMO or Mg-doped LNMO), polyvinylidene difluoride (PVDF) and acetylene black were mixed in the N-methyl-2-pyrrolidone (NMP) solution at a weight ratio of 8:1:1 to form a uniform slurry. The homogeneous slurry was printed onto aluminum foil using a doctor blade and dried in a vacuum oven at 120 °C for 12 h to remove the solvent. When the temperature dropped to 25 °C, the dried electrodes were pressed and cut into circles with a diameter of 12 mm. The final mass of active material per individual electrode is approximately 1.2 to 1.7 mg. Half-cell (CR2032-type coin cell) assembling was carried out in an MIKROUNA glovebox with the moisture and oxygen level below 0.01 ppm. Lithium foil and Celgard 2500 polypropylene film were employed as the counter electrode and separator, respectively. The electrolyte solution was composed of 1 M LiPF_6_ in a 3:7 volume ratio mixture of ethylene carbonate (EC) and methyl ethyl carbonate (EMC). Charge/discharge testing was carried out at 3.5–4.9 V using a CT2001A Land battery testing system at room temperature. Cyclic voltammetry (CV) was performed on an electrochemical workstation (CHI606E) at a scan rate of 0.01 mV s^−1^ between 3.5 V and 4.9 V. Electrochemical impedance spectroscopy (EIS) measurements were performed at 3.5 V on the same instrument with a disturbance amplitude of 5 mV between 100 kHz and 10 mHz.

## 3. Results and Discussion

### 3.1. Structure and Morphology

**Figure 1:**
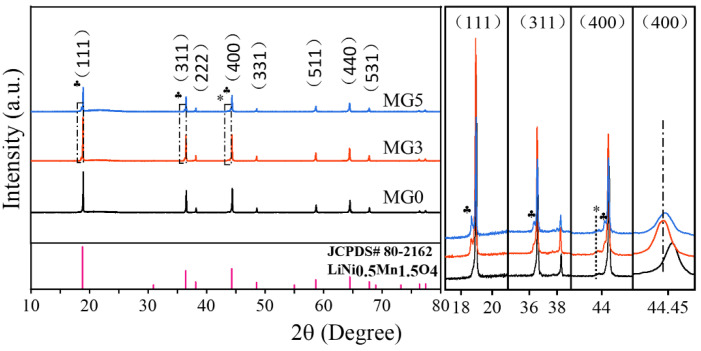
XRD patterns of three samples with the enlarged detail of the dotted box; Symbol * represents Li_x_Ni_1−x_O impurity; Symbol ♣ represents 16c sites.

The XRD patterns of the materials LiNi_0.5_Mg_x_Mn_1.5−x_O_4_ (x = 0, 0.03, 0.05) are shown in Figure 1. It is seen that the typical profiles of the spinel phase (JCPDS No. 80-2162) with the Fd-3m cubic space group are displayed, and the peaks are narrow and sharp with different Mg doping. Obviously, MG3 is the strongest among them, indicating the best crystallinity. From the position of 44.4° peak (400), two doped samples show a slight shift to smaller angles compared to the undoped MG0, with the maximum shift observed in MG3. It is mainly due to Mg^2+^ (0.72 Å) having the largest ionic radius among Ni^2+^ (0.69 Å), Mn^3+^ (0.65 Å) and Mn^4+^ (0.53 Å) [20,25]. And it also means Mg enters the LNMO lattice well when x < 0.03 and reaches the maximum saturation when x = 0.03. In the three dotted boxes on the right of Figure 1, it can be found that the peaks appear at 18.65°, 36.3° and 44.2° positions (marked with ♣) in the MG3 sample, with a visible increase in peak intensity with the addition of Mg (MG5). It can be attributed to the transition or alkali metal (Mn, Ni or Mg) occupancy at the interstitial 16c sites [12,13,17,18,19]. And such situations have never been reported in the modification of Mg-Mn hetero-valent doping in the past, even with a high doping amount about LiNi_0.5_Mg_0.1_Mn_1.4_O_4_ [23,24]. The strategy of Microwave sol-gel method and Mg-Mn hetero-valent substitution can together lead to the interstitial 16c sites modification. In addition, there is still a negligible Li_x_Ni_1−x_O impurity at the position of the 43.8° position (marked with *), which is mainly due to the undesired second phases from oxygen loss at a high sintering temperature [26].

**Figure 2:**
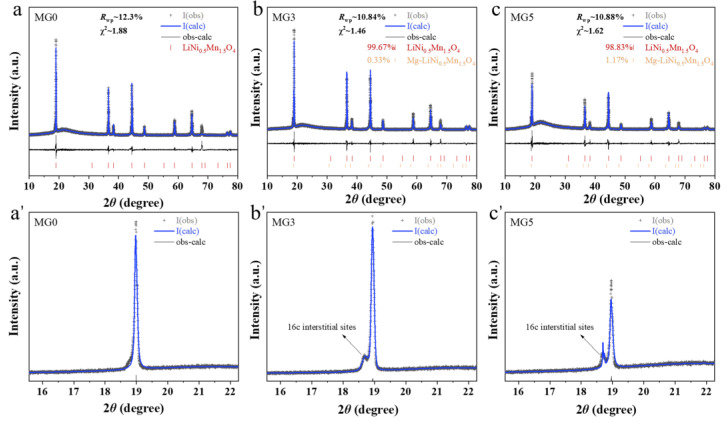
Refined XRD results of (**a**,**a’**) MG0, (**b**,**b’**) MG3 and (**c**,**c’**) MG5 samples.

The further refinement results are shown in Figure 2. Before the calculations, LNMO containing 0.1 Mg (16c) was used as the reference phase for the 16c site, designated as Mg-LNMO [19]. The final fitting yielded reliable results, with Rwp values of 12.3%, 10.84% and 10.88% for Mg0, Mg3 and Mg5, respectively. The detailed (111) plane image also confirms a high degree of fitting accuracy. Additional refined composition and crystallography details can be found in Table S1. The results indicate that as the Mg doping level increases, the proportion of Mg-LNMO also rises, with Mg5 exhibiting a Mg-LNMO content of 1.17%. This implies that the Mg5 sample has a higher occupancy at the 16c site.

**Figure 3:**
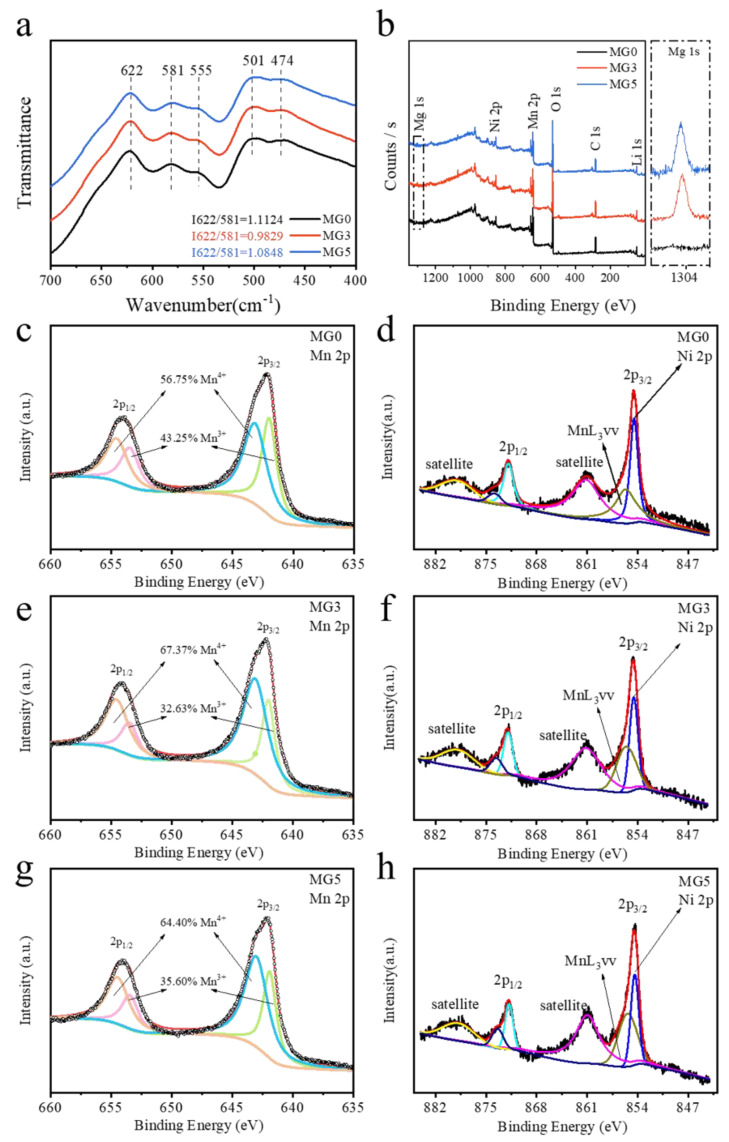
(**a**) FTIR spectra; (**b**) XPS spectrum of the samples and the magnification is the Mg peak (the part of the dotted box); Mn 2p and Ni 2p spectra of (**c**,**d**) MG0, (**e**,**f**) MG3 and (**g**,**h**) MG5.

Figure 3a shows the IR absorption of all samples in the frequency range from 400 to 700 cm^−1^. Depending on the distribution of Mn and Ni ions in the lattice, LiNi_0.5_Mn_1.5_O_4_ has two different crystallographic structures: one is in the Fd-3m space group with a random distribution of Ni and Mn ions at the 16d octahedral sites, and the other is in the P4_3_32 space group with the Ni and Mn ions ordered at the octahedral 4b and 12d sites [27]. Owing to the similar scattering factors of Ni and Mn ions, the phase of LiNi_0.5_Mn_1.5_O_4_ could not be accurately identified by XRD analysis. FTIR spectroscopy was performed to further differentiate the ordered/disordered structure of the LiNi_0.5_Mn_1.5_O_4_. It can be seen from picture that the three curves are similar and five obvious IR absorption locates at 622, 581, 555, 501 and 474 cm^−1^, which belongs to Fd-3m structure [27,28,29]. This is consistent with the XRD results and further confirms that all samples are still from the disordered Fd-3m space group. Mg doping did not change the crystal structure of three sample. Moreover, the intensity ratio between the bands 622 and 581 cm^−1^ can be used to assess qualitatively the Ni/Mn disordering degree in the spinel lattice [28]. The relevant results are 1.1124, 0.9829 and 1.0848 (MG0, MG3 and MG5), respectively. This shows that the introduction of Mg will lead to a decrease in the disorder of Mn/Ni, which is consistent with the results of previous simulation reports in the literature [30]. At the same time, the minimum disordering degree of MG3 also proves that the upper limit of Mg doping is 0.03.

Normally, the chemical state has a great influence on the crystal structure and electrochemical performances. Hence, XPS was conducted to figure out the surface structure of the samples in Figure 3. The elemental composition of spinel LNMO and doped material was found in the full spectrum of XPS, O 1s, Mg 1s, Li 1s, Mn 2p and Ni 2p in Figure 3b. The peak of the Mg 1 s (1303.3 eV) region was clearly enlarged in the right image. It can be seen that MG0 has no obvious Mg peak, while clear Mg signals appear in doped samples. As shown in Figure 3c,d, the peaks at 642.0 and 643.1 eV correspond to Mn^3+^ and Mn^4+^ in the Mn 2p_3/2_ spectrum of MG0, respectively. The introduction of Mg effectively reduced the content of Mn^3+^ from 43.25% (Mg0) to 32.63% (Mg3). However, this decrease is limited, with MG5 exhibiting rising content of 35.6%. It should be noted that XPS, as a surface testing method, cannot represent the Mn^3+^ contents of the entire bulk phase, and such results are suggested to be qualitative evidence. Electrochemical quantification in the 4 V plateau, as a bulk quantitative measurement result, can provide a good estimate for the Mn^3+^ concentration in the whole LNMO materials, which will be discussed later [31]. The binding energy at 854.5 and 871.9 eV belongs to Ni2p_3/2_ and Ni2p_1/2_ of MG0, respectively. And the satellite peak was located at 861.2 eV, all of which represents a typical of Ni2p spectrum. In addition, the binding energy at 855.75 eV is the MnLVV Auger transition with Alk excitation, which is quite near to the peak of Ni2p_3/2_ but not Ni^3+^ [32].

**Figure 4:**
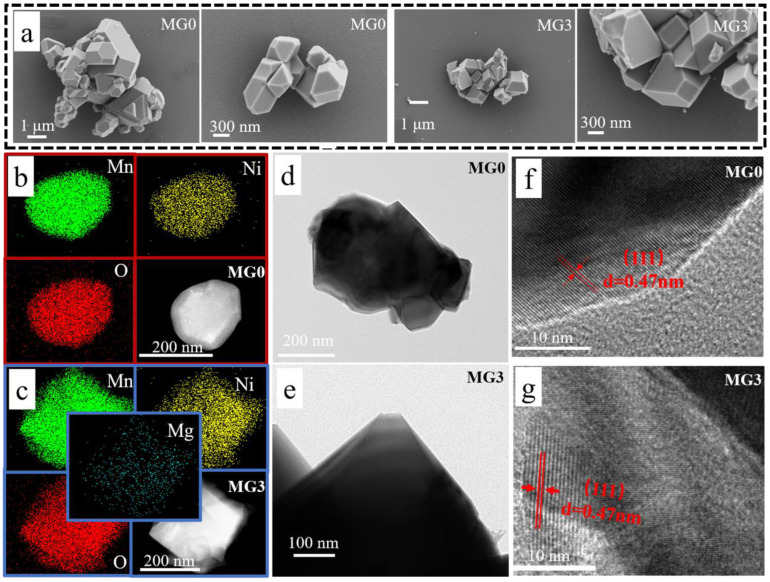
(**a**) SEM images of the samples; elemental mapping for Mn, Ni, Mg and O of the (**b**) MG0 and (**c**) MG3 particles; TEM and corresponding high-resolution images of (**d**,**f**) MG0 and (**e**,**g**) MG3.

The morphology and particle size of all the samples were investigated by scanning electron microscopy (SEM) images and transmission electron microscopy (TEM) shown in Figure 4. As seen in Figure 4a, all the samples synthesized by microwave-assisted sol-gel method are composed of nano and micro-sized particles with a regular truncated octahedral shape. Compared with the common octahedral spinel particles composed entirely of (111) surfaces, the truncating large portions of (100) surfaces can stabilize the spinel structure and support Li^+^ transport kinetics [33]. Energy dispersive spectrometry (EDS) mapping analysis of MG0 and MG3 shown in Figure 4b,c. The elements of Ni, Mg and Mn are homogeneously distributed throughout the particle without any agglomerated phases arising. The transmission electron microscopy (TEM) images shown in Figure 4d (MG0) and 4e (MG3) further illustrate the polyhedral structures with smooth planed and well-defined edges of the samples. In addition, the surface of the particles was further examined by high resolution transmission electron microscopy (HRTEM). Both the clear crystal planes are shown in Figure 4f (MG0) and 4g (MG3) with a d-spacing of 0.47 nm corresponding to the (111) planes. The related MG5 data are shown in Figure S2, which exhibits similar morphology and d-spacing.

### 3.2. Electrochemical Characterization

**Figure 5:**
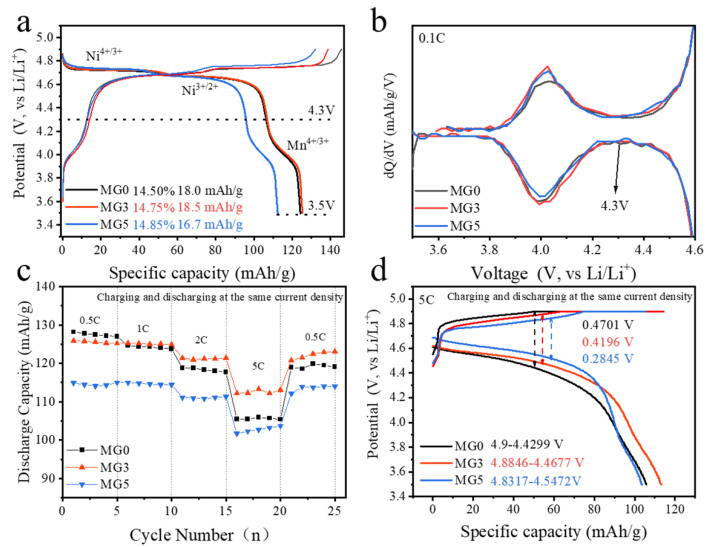
(**a**) The discharge curves of three samples during the second cycle collected at 0.1 C between 3.5 and 4.9 V (vs. Li^+^/Li), and (**b**) dQ dV^−1^ profiles at 0.1 C in 4.0 V region; (**c**) rate-capability test and (**d**) the corresponding charge and discharge profile at 5 C.

The electrochemical performance of the samples was investigated in coin-half cells using metallic lithium foil as counter electrodes. Figure 5a shows the discharge curves of the three samples between 3.5 and 4.9 V at a current density of 0.1 C (1 C = 147 mAh g^−1^) at 25 °C. Three discharge platforms in the 4 V and 4.7 V regions indicate that all samples belong to the disordered LNMO (Fd-3m) space group. From the corresponding dQ dV^−1^ result in Figure 5b, the capacity in 3.5–4.3 V region can be provided by Mn^4+/3+^ redox pairs [34]. It can be observed that Mg substitution with Mn can reduce the content of Mn^3+^ (Mg5, 16.7 mAh/g), but the effect is limited compared to unmodified MG0 (18 mAh/g). Moreover, the increasing doping amount will reduce the initial discharge specific capacity. MG5 just delivered a specific discharge capacity of about 112.2 at 0.1 C, while MGO had 124.2 mAh/g. Therefore, the final Mn^3+^ proportion of three samples is approximately 14.5%.

The rate performances of the samples are demonstrated in Figure 5 and Figure S3. MG3 can liberate a capacity of about 113.1 mAh/g, while the MG0 is 105.4 mAh/g at 5 C. The corresponding rate retention (5 C/0.5 C) is 82.60 and 89.99%. This may be due to the larger ionic radius of Mg^2^⁺, which increases the transport channels for lithium ions and effectively enhances their diffusion capability. This is consistent with the XRD data and its refinement results, indicating that the introduction of Mg enlarges the lattice constant of LNMO [20,35,36]. Interestingly, MG5 also has a high retention rate of 90.25%. After the rate test, all samples are charged and discharged back to 0.5 C; MG3 and MG5 still maintain excellent rebound capacity, indicating that Mg doping can availably strengthen the rate performance. It may be attributed to the 16c occupancy improving the stability of the structure [12,13,18]. As shown in Figure 5d, the 5 C charge–discharge curves show that MG5 has the smallest average voltage difference about 0.2845 V, indicating a potential for improved cycle stability during the 5 C charge and discharge process. The relevant lithium diffusion coefficient of three samples were shown in Figure S8 and Table S3, which further illustrates that Mg doping facilitates lithium transport [37,38].

**Figure 6:**
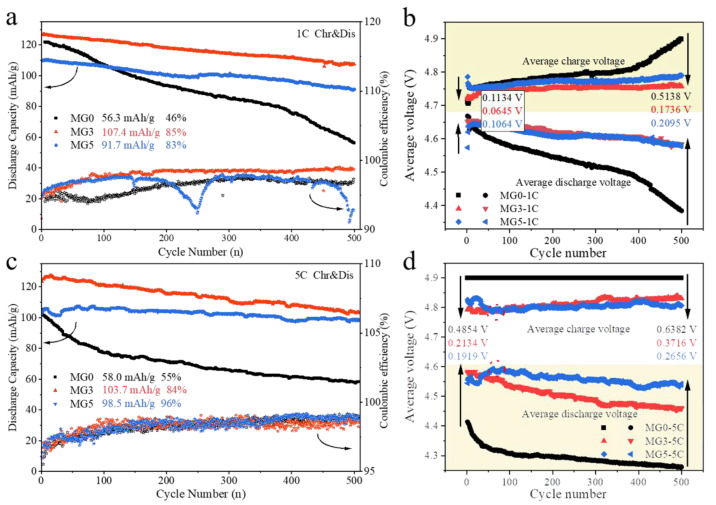
The cycle performances of the samples at current density of (**a**) 1 C and (**c**) 5 C for 500 cycles at 25 °C. Corresponding average charge–discharge voltage profiles at (**b**) 1 C and (**d**) 5 C.

**Figure 7:**
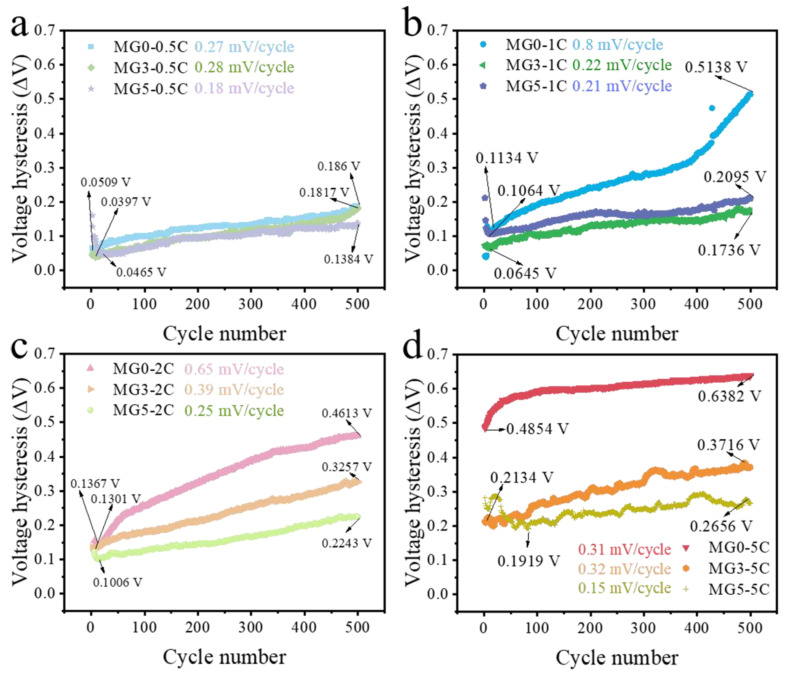
The voltage hysteresis from average charge–discharge voltage profiles of three samples (MG0, MG3 and MG5) at different current densities: (**a**) 0.5 C, (**b**) 1 C, (**c**) 2 C and (**d**) 5 C.

The long cycle performances were tested at different charge and discharge current density (0.5–5 C), shown in Figure 6a and Figure S5. At 1 C cycling, the undoped sample MG0 dropped rapidly with a value of discharge capacity of about 56.3 mAh g^−1^ (46% retention to initial value) after 500 cycles, while MG3 and MG5 present good cyclic stability (85% and 83%). From Figure 6b, the higher average charge voltage and lower discharge voltage of MG0 indicate the serious polarization during the cycling [39]. In contrast, the stable average charge–discharge voltage of MG3 and MG5 with lowered voltage hysteresis (0.22 and 0.21 mV/cycle; Figure 7b) were measured at 1 C, remarkably better than that of undoped MG0 (0.8 mV/cycle). The relevant calculations are described in the literature [39] and Figure S4. In simple terms, the voltage hysteresis is defined as the voltage difference between the average plateau voltages during charge and discharge within a single cycle. After 500 cycles, the voltage hysteresis for each cycle was measured. To determine the incremental average hysteresis per cycle throughout the cycling process, the average difference between the voltage hysteresis of the 500th cycle and that of the initial cycle was calculated, as shown in Figure 7. A more stable cycling system implies a smaller single-cycle polarization, which is visually manifested as a smaller increase in voltage hysteresis. These results establish that the introduction of Mg can effectively stabilize the cyclic performance of LNMO.

At a higher current density of 5 C, the difference between the average charge and discharge voltages for MG0 becomes more pronounced (Figure 6d). Additionally, the average discharge voltages of MG3 are less stable compared to those at 1 C (Figure 6b). In contrast, MG5 exhibits lower voltage hysteresis, approximately 0.15 mV/cycle (Figure 7d). This suggests that increased Mg-Mn doping is crucial for the stability of high-rate cycling performance. However, this hetero-valent doping strategy negatively impacts capacity. Although MG3 has a lower capacity retention rate (84%) compared to MG5 (96%), its higher initial discharge capacity results in the highest remaining specific capacity (103.7 mAh g^−1^) after 5 C cycling (Figure 6c). This allows for greater flexibility in application depending on the requirements, such as using MG3 for applications with high capacity demands or MG5 for those requiring stable high-rate cycling. Additional cycling data at different current densities (0.5 C and 2 C) are provided in Figure S5. The lower voltage hysteresis of MG5 directly manifests as a higher degree of overlap in the discharge profiles during cycling, indicating more stable cycling performance (Figure S6f).

**Figure 8:**
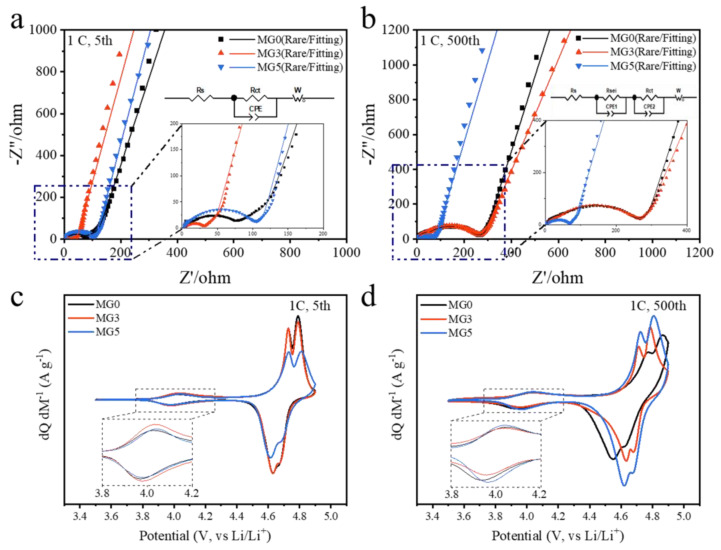
Nyquist plots of all samples curves after (**a**) 5 and (**b**) 500 cycles at 1 C and state of 3.5 V with the rare and fitting results, and the insert is the magnification of the high frequency region and the equivalent circuit performed to fit the curves; the Cyclic voltammograms at a scan rate of 0.1 mV s^−1^ after (**c**) 5 and (**d**) 500 cycles at 1 C.

Electrochemical impedance spectroscopy (EIS) and Cyclic voltammograms (CV) measurements were selected to further detect the cycle performance of three samples. As shown in Figure 8a, all fresh samples consist of one semicircle in the high-medium frequency region and a sloping line at low frequency region. The corresponding equivalent circuit was obtained by the Zview Microsoft (v3.1) simulation (Figure 8a; inset). In general, the intercept in high frequency region was related to the cell bulk resistance (Rs), reflecting the electric conductivity of the electrolyte, separator and electrodes. Subsequently, a semicircle at high frequencies corresponds to the charge transfer resistance (Rct) and interfacial capacitance at the electrode/electrolyte interface (CPEct). At the end of the plots, a straight line in low frequency region assigned to Warburg impedance (W), which is controlled by the solid-state diffusion of Li^+^ ions in the bulk of intercalation compound (namely, a simplified equivalent circuit model: Rs−Rct/CPEct−W) [33]. A more detailed comparison is provided in Figure S7. The Rct of the MG3 is 23.12 Ω excellently smaller than others before a long cycling. Nevertheless, it is interesting that the Rct of MG0 and MG3 increased to 210.5 and 213.2 Ω after cycling, while MG5 decreased to only 51.74 Ω. This may be due to an activation process that occurs during the initial cycles of the battery, which results in a gradual decrease in impedance until it stabilizes [40]. During further cycling, factors such as electrolyte decomposition, electrode material shedding and dissolution of the positive electrode material can cause an increase in impedance [41,42]. This is reflected in the increase in voltage hysteresis and the reduction in capacity. Therefore, the stability of the impedance for MG5 is consistent with its cycling performance. Both observations suggest that the Mg-Mn hetero-valent doping and the 16c interstitial sites contribute to the improvement of cycling performance.

It can be seen in Figure 8c that all samples exhibited three typical reversible peaks of disordered LNMO (Fd-3m) material. A pair of minor peaks owing to the Mn^3+^/Mn^4+^ couple can be observed around 4 V region. Two well-separated peaks in the high voltage from 4.6 to 4.8 V are corresponding to Ni^2+^/Ni^3+^ and Ni^3+^/Ni^4+^ redox couples, which indicates a two-stage Li^+^ extraction/insertion from/into the spinel framework. As shown in Figure S7d, the oxidation peak (O2) of MG0 shifted to the higher potential (ΔO = Δ5th − Δ500th = −0.076 V), and the reduction peak (R1) shifted to the lower potential (ΔR = Δ5th − Δ500th = 0.085 V) after cycling, which are consistent with the results of average voltage analysis in Figure 6b. It indicated a decrease in the cycling reversibility of MG0. After doping modification, both MG3 and MG5 exhibited the stable oxidation-reduction peak shifts. This, along with the EIS analysis, indicates that Mg-Mn hetero-valent doping and the 16c interstitial sites contribute to the enhancement of cycling performance.

## 4. Conclusions

In this work, a hetero-valent Mg-Mn substitution LiNi_0.5_Mg_x_Mn_1.5−x_O_4_ (x = 0, 0.03, 0.05) was synthesized via a microwave sol-gel method. This strategy has resulted in a 16c modification that was not observed with previous Mg-Mn hetero-valent doping. As a result, the cycling stability of the doped samples is significantly enhanced, with the capacity retention after 500 cycles at 1 C improving from 46% for the undoped sample to 85% (MG3) and 83% (MG5). At higher cycling rates 5 C, the MG5 sample with more extensive 16c modification exhibits even more stable cycling performance, maintaining a 96% capacity retention after 500 cycles. The voltage hysteresis is only 0.15 mV/cycle, further demonstrating the positive impact of hetero-valent doping on cycling stability. However, given the relatively low load used in this study, future applications may require testing with larger loads and full battery assessments to validate these improvements. Additionally, LNMO with varying doping levels offers tailored solutions for various application needs, including capacity-focused or high-current-rate applications for MG3 and MG5. Furthermore, the stable, higher substitution sample MG5 could also serve as an effective coating layer for other electrode materials to enhance cycling stability.

## Data Availability

The original contributions presented in the study are included in the article/Supplementary Material, further inquiries can be directed to the corresponding author.

## Supplementary Materials

The following supporting information can be downloaded at: https://www.mdpi.com/article/10.3390/ma17194714/s1, Scheme S1: The process of synthesizing the LiNi0.5MgxMn1.5-xO4 samples; Figure S1: Detailed picture of microwave sol-gel reaction equipment; Figure S2: SEM and TEM images of MG5; Figure S3: The rate capacity ratio of three samples; Figure S4: The voltage hysteresis of MG3 at 10th cycle at 1C; Figure S5: The cycle performances of the samples at current density of (a) 0.5C and (b) 2C for 500 cycles at 25 °C; Figure S6: The discharge curves under different cycles of (a,d) MG0, (b,e) MG3 and (c,f) MG5 at 1C and 5C; Figure S7: The EIS and CV comparison results of (a,d) MG0, (b,e) MG3 and (c,f) MG5; Figure S8: Cyclic voltammograms at various scan rate in the potential range from 3.5 to 4.9 V vs. Li/Li+ of the (a–e) MG0 to MG7 electrode; Table S1: Phase composition and crystallography details of LNMO and Mg0.1-LNMO obtained from refined XRD results; Table S2: XPS data of three samples; Table S3: Apparent Lithium Diffusion Coefficients (DLi) calculated from CV measurements for the all LNMO materials [37,38].
