# Supplementary material for: Enhanced Cycling Performance of Spinel LiNi0.5Mn1.5O4 Cathodes through Mg-Mn Hetero-Valent Doping via Microwave Sol-Gel Method
Source: Materials (Basel). 2024 Sep 25;17(19):4714. doi: 10.3390/ma17194714 (PMC11477552; doi:10.3390/ma17194714)
Supplement: Supplementary file 1 [file materials-17-04714-s001.zip › materials-3218376-supplementary.pdf]

Enhanced Cycling Performance of Spinel $LiNi_{0.5}Mn_{1.5}O_4$ Cathodes through Mg-Mn Hetero-valent Doping via Microwave Sol-Gel Method

Mingyin Su [1], Xiongwen Dong [2], Xinyi Dai [1], Bingbing Huang [1], Min Shen [1], Teng Xu [1] and Qibin Liu [1,3,*]

[1] College of Material and Metallurgy, Guizhou University, Guiyang 550025, China
[2] Guizhou Dalong Huicheng New Material Co., Ltd., Tongren 557503, China
[3] Key Laboratory of Modern Manufacturing Technology of Educational Ministry, Guiyang 550025, China
* Correspondence: qbliugzu@163.com

1. Supported Figure

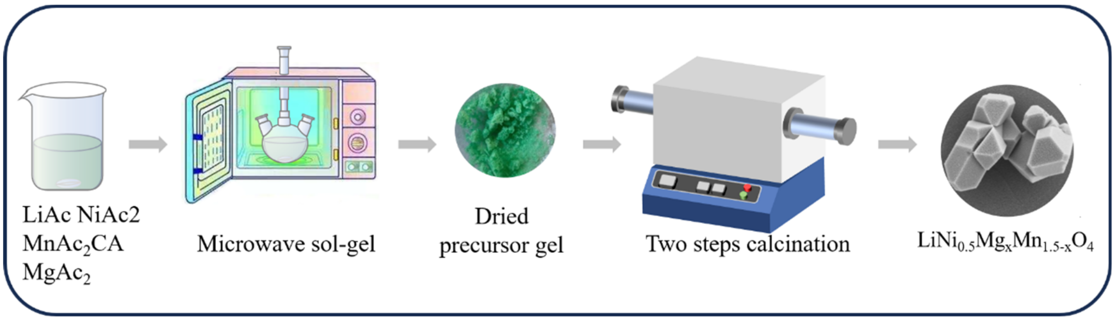

Scheme S1. The process of synthesizing the $LiNi_{0.5}Mg_xMn_{1.5-x}O_4$ samples.

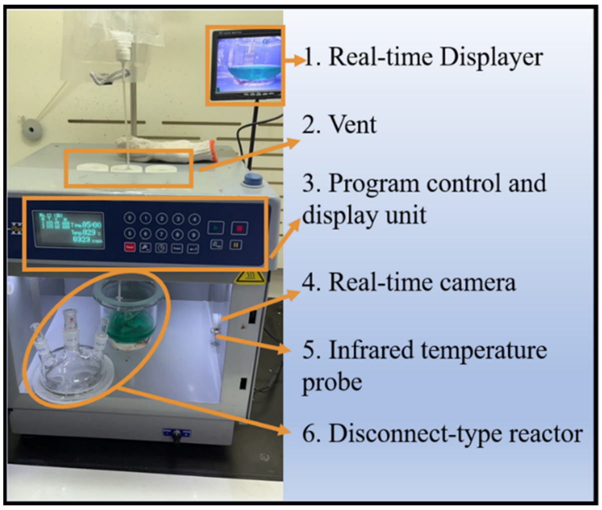

Figure S1. Detailed picture of microwave sol−gel reaction equipment.

This is a microwave reaction device, which primarily includes an internal microwave generator, a program control section (3), and a temperature detection device (5). During the reaction process, we can monitor the real−time status of the reaction through the camera (4) and the screen (1). In simple terms, its principle is similar to that of a household microwave oven. The difference is that additional modules have been introduced to make the reaction power, reaction temperature, and reaction time controllable and observable.

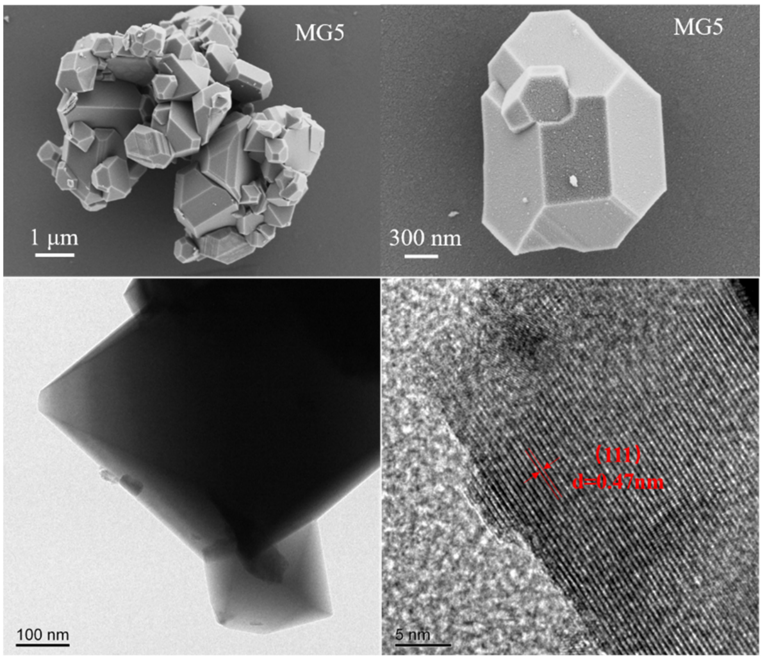

Figure S2. SEM and TEM images of MG5.

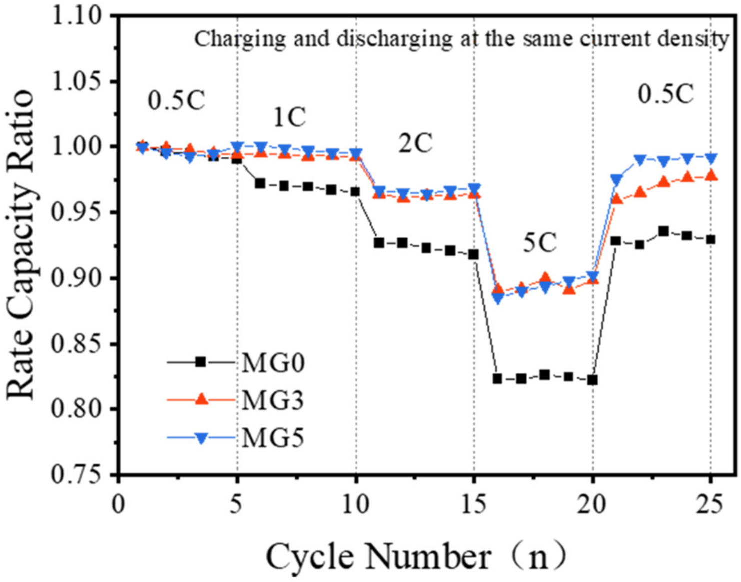

Figure S3. The rate capacity ratio of three samples;.

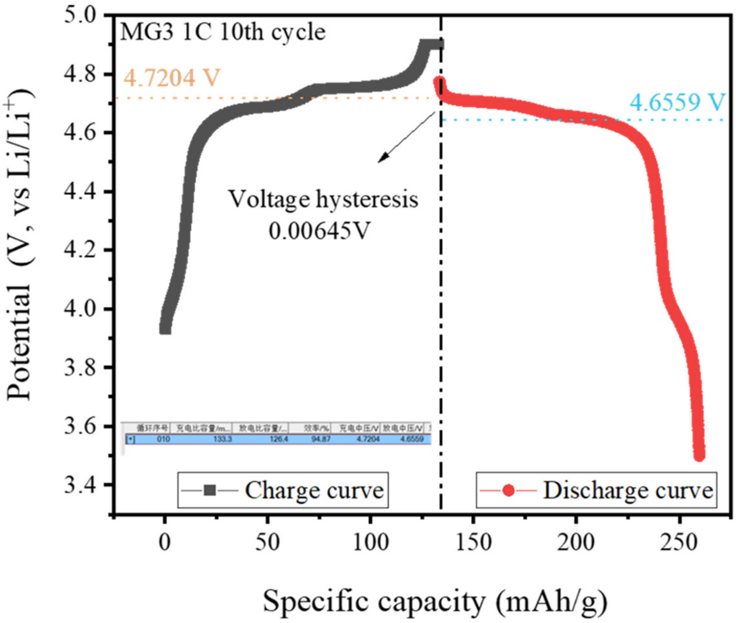

Figure S4. The voltage hysteresis of MG3 at 10th cycle at 1C.

The voltage hysteresis is obtained from cycling data. And it is defined as the voltage difference between the average plateau voltages during charge and discharge within a single cycle. The voltage hysteresis of a cyclic process is illustrated in the following diagram. After 500 cycles, the voltage hysteresis for all cycles was obtained, as shown in Figure 5b and 5d. Subsequently, taking the average difference between the voltage hysteresis at the 500th and initial cycle yields the incremental average hysteresis per cycle during the cycling process, as shown in Figure 6.

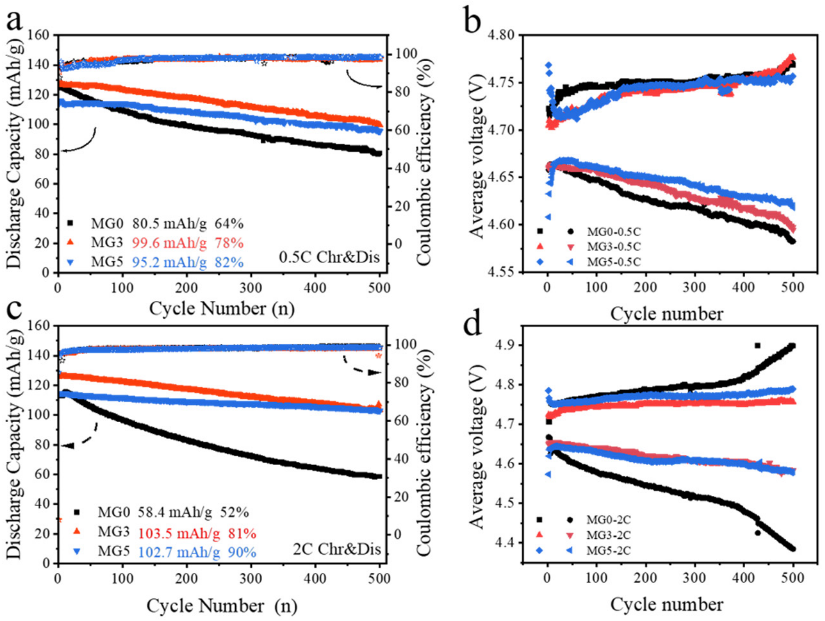

Figure S5. The cycle performances of the samples at current density of (a) 0.5C and (b) 2C for 500 cycles at 25 °C. Corresponding average charge–discharge voltage profiles at (c) 0.5C and (d) 2C.

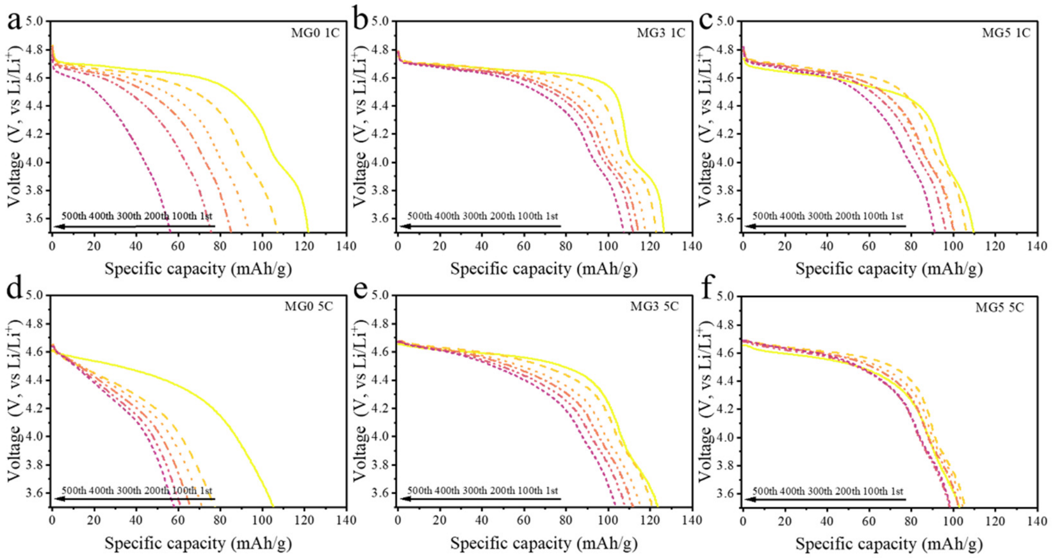

Figure S6. The discharge curves under different cycles of (a,d) MG0, (b,e) MG3 and (c,f) MG5 at 1C and 5C;.

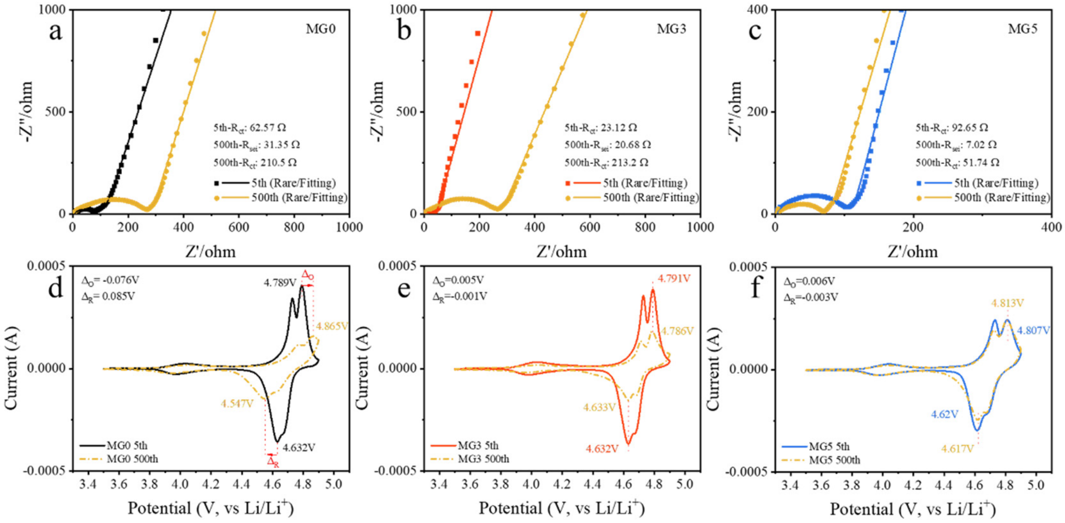

Figure S7. The EIS and CV comparison results of (a,d) MG0, (b,e) MG3 and (c,f) MG5.

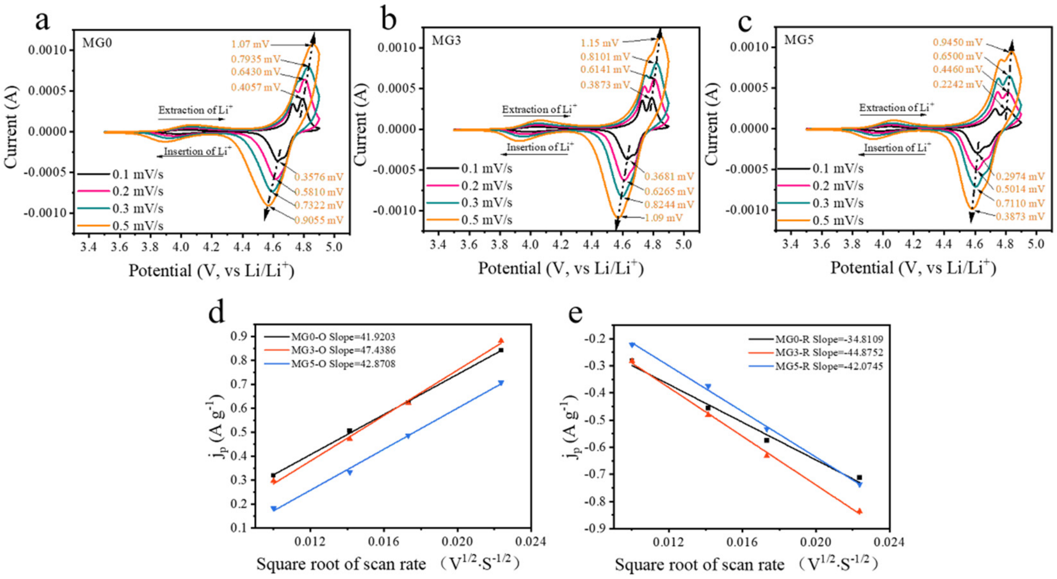

Figure S8. Cyclic voltammograms at various scan rate in the potential range from 3.5 to 4.9 V vs. Li/Li^+ of the (a–e) MG0 to MG7 electrode; The five samples corresponding linear response of the (f) anodic and (g) cathodic peak current density as a function of the square root of the scan rate;.

Figure S8 shows that the CVs recorded at different voltage scanning rates of samples. Assuming that the electrochemical reaction rate is limited by the solid–state diffusion of Li^+, the peak current and the CV scan rate satisfy the following Randles–Sevcik equation [1, 2]:

$$j_p = 2.69 \times 10^5 \, n^{\frac{3}{2}} \, A \, \Delta C_0 \, D_{Li}^{\frac{1}{2}} \, v^{\frac{1}{2}} \quad (2)$$

In the above equation, j_p stands for the peak current density (A g^{-1}), n stands for the number of transfer electrons per reaction species (for Li^+ n = 1), A stands for the apparent area of the electrode (1.13/m cm^2 g^{-1}, m here is the mass of active materials $LiNi_{0.5}Mn_{1.5}O_4$), ΔC_0 stands for the bulk concentration of Li^+ (0.02378 mol cm^{-3}), D_{Li} stands for the diffusion coefficient (cm^2 s^{-1}) and v stands for the potential scan rate (V s^{-1}). In the present case, as shown in Figure S8g and S8f, j_p is proportional to $v^{1/2}$ confirming a diffusion–controlled behavior. From the slope of the linear fit, we have calculated the apparent diffusion coefficients of all materials, which are listed in Table S3. Furthermore, it is evident that the kinetics of Li^+ diffusion for MG3 electrode presents the optimum average diffusion coefficient D_{Li} of 6.94×10^{-11} cm^2 s^{-1}.

2. Supported Table

Table S1. Phase composition and crystallography details of LNMO and Mg0.1–LNMO obtained from refined XRD results.

	MGO	MG3	MG5
LNMO	1	99.67%	98.83%
LNMO–Mg	0	0.33%	1.17%
a (Å)	8.1746	8.1778	8.1831
Volume	546.2602	546.9019	547.966

LiNi0.5Mn1.5O4 (LNMO)

Space group: F d −3 m

Atom	Wyckoff site	x	y	z	frac	Uiso
O	32e	0.1382	0.1382	0.1382	1	0.01
Li	8a	0	0	0	1	0.01
Mn	16d	0.375	0.375	0.375	0.75	0.01

Atom	Wyckoff site	x	y	z	frac	Uiso
Ni		0.375	0.375	0.375	0.25	0.01
$LiNi_{0.5}Mn_{1.5}O_4$−$Mg_{0.1}$ (LNMO−Mg)<br>Space group: F d −3 m						
Atom	Wyckoff site	x	y	z	frac	Uiso
O	32e	0.1382	0.1382	0.1382	1	0.01
Li	8a	0	0	0	1	0.01
Mn	16d	0.375	0.375	0.375	0.75	0.01
Ni		0.375	0.375	0.375	0.25	0.01
Mg	16c	−0.125	−0.125	−0.125	0.1	0.01

Table S2. XPS data of three samples.

		**Peak position**	**FWHM**	**Valence**	**Peak intensity**	**Percentage**
MG0	2p3/2	641.98	1.47	+3	68265.82	0.4325
		643.14	2.32	+4	89564.11	0.5675
	2p1/2	653.47	1.81	+3	34132.91	0.4325
		654.56	2.16	+4	44782.05	0.5675
MG3	2p3/2	642.00	1.32	+3	46372.46	0.3263
		643.09	1.70	+4	95753.95	0.6737
	2p1/2	653.49	2.43	+3	23186.23	0.3263
		654.53	2.28	+4	47876.98	0.6737
MG5	2p3/2	641.90	1.31	+3	46886.45	0.3560
		643.01	2.27	+4	84834.70	0.6440
	2p1/2	653.44	1.66	+3	23443.22	0.3560
		654.45	2.19	+4	42417.35	0.6440

Table S3. Apparent Lithium Diffusion Coefficients (D_{Li}) calculated from CV measurements for the all LNMO materials.

	**D_{Li} ($\times 10^{-11}$ cm^2 s^{-1})**		
	O−D_{Li}	***R−D_{Li}***	**D_{Li}**
MG0	5.44	3.75	4.60
MG1	5.23	4.13	4.68
MG3	7.32	6.55	6.94
MG5	6.28	6.05	6.17
MG7	4.86	5.14	5.00

O−D_{Li}, O−D_{Li} and D_{Li} represent the D_{Li} of oxidation, D_{Li} of reduction and the average value of O−D_{Li} and O−D_{Li}, respectively.

Reference

1. Liu, H.; Wang, J.; Zhang, X.; Zhou, D.; Qi, X.; Qiu, B.; Fang, J.; Kloepsch, R.; Schumacher, G.; Liu, Z.; Li, J., Morphological Evolution of High−Voltage Spinel LiNi(0.5)Mn(1.5)O4 Cathode Materials for Lithium−Ion Batteries: The Critical Effects of Surface Orientations and Particle Size. *ACS Appl Mater Interfaces* **2016,** 8, (7), 4661−75.
2. Jiang, C.; Tang, Z.; Wang, S.; Zhang, Z., A truncated octahedral spinel LiMn 2 O 4 as high−performance cathode material for ultrafast and long−life lithium−ion batteries. *Journal of Power Sources* **2017,** 357, 144−148.
